# Evidence asymmetry in Australian public spending: why health alone cannot bear a higher burden of proof

**DOI:** 10.3389/fpubh.2026.1843884

**Published:** 2026-05-20

**Authors:** Leonard Lee, Joshua Byrnes, Mark Stewart, Martin Snoke, Hansoo Kim

**Affiliations:** 1 School of Medicine and Dentistry, Griffith Health, Griffith University, Birtinya, QLD, Australia; 2 Roche Products Pty Ltd., Sydney, NSW, Australia; 3 Bond University, Gold Coast, QLD, Australia; 4 Griffith Asia Institute, Nathan, QLD, Australia

**Keywords:** Australia, federal budget, health policy evaluation, public sector governance (PSG), social return on investment (SROI)

## Abstract

Australian Commonwealth portfolio expenditure operates under structurally asymmetric evidentiary standards that disproportionately burden the Health portfolio and distort cross-portfolio funding decisions. Despite comparable fiscal scale, Health (AU$70.8 billion projected for 2025–26) is subject to rigorous formal evaluation through mechanisms such as the Pharmaceutical Benefits Advisory Committee and Medical Services Advisory Committee, while portfolios including Education (AU$72.5 billion) and Defence (AU$83.2 billion) justify expenditure predominantly through strategic alignment, narrative reporting, and performance indicators without reference to validated methodological benchmarks. This creates a paradox in which Health face greater barriers to funding than those relying on informal justificatory approaches, limiting the ability of central agencies to make transparent, comparable, and welfare-maximising funding decisions. Drawing on cross-portfolio analysis of Commonwealth Budget papers, portfolio budget statements, and central agency guidance documents, this commentary demonstrates that this asymmetry is structural rather than incidental, arising from inconsistently applied evaluative expectations that undermine efficient allocation of public resources. The Federal Government’s Measuring What Matters framework explicitly endorses broader conceptions of value including wellbeing and equity, yet these remain absent from Expenditure Review Committee funding decisions, widening the gap between stated policy priorities and practice. Social Return on Investment is proposed as one potential pragmatic bridging framework capable of extending proportional evaluative standards across portfolios currently assessed through informal mechanisms, complementing established health economic methods. Aligning evidentiary standards with expenditure magnitude and societal impact requires institutional reform that makes wellbeing assessable, comparable, and consequential within budget processes, with Health well positioned to lead as a proof of concept.

## Introduction

Australian public spending operates under a structural paradox. Portfolios of comparable fiscal scale face markedly different expectations regarding how they justify value, effectiveness, and impact. The Health portfolio (Health) has been projected to spend approximately AU$70.8 billion for 2025–26, with Education projecting approximately AU$72.5 billion (1). Despite the similarities in fiscal weighting, Health must meet high evidentiary standards through formal economic appraisal, structured outcome reporting, and institutionalised review pathways, through mechanisms such as PBAC, MSAC, and long-standing outcome-based performance reporting (2). In contrast, Education relies predominantly on program logic, narrative justification, performance indicators, and statutory oversight mechanisms without reference to validated methodological benchmarks (Table 1) (1).

**Table 1:** Comparison of domestic portfolios^a^.

Portfolio	Quality of evidence	Evaluation methods	External variables	Portfolio size 2025–2026 (AUD)	Key programs/outcomes
Defence	Indirect only (strategy documents and performance targets with no reference to evidence standards)	Informal: strategic planning, performance indicators, National Defence Strategy cycle	Geopolitical risk (AUKUS, Indo-Pacific tensions), national security	$83.2B	Defence operations, submarine agency, housing
Health and Aged Care	Explicitly states use of “evidence-based health policy,” with outcomes tied to measurable objectives	Formal: evidence-based policy and performance metrics across timeframes	Aged care reforms, ageing demographic	$70.8B	Medicare, PBS
Social Services	A balance between programs with explicit evidence standards, and others without.	A mix of formal and informal: evidence-based policy and performance metrics, and informal stakeholder engagement.	Strongly shaped by demographic and socioeconomic trends (disability reform, housing, domestic/family violence, Aboriginal and Torres Strait Islander priorities)	$254.3B	NDIS, social security, housing, disability/carer support, Services Australia delivery
Infrastructure	No explicit mention of evidence standards	Informal: performance indicators, stakeholder surveys	Climate change, regional equity	$14.8B	Emergency services, infrastructure investment, communications
Education	Indirect only via performance measures and statutory agency oversight	Informal: performance indicators, progress reports, stakeholder feedback	Access and equity issues, workforce needs, digital literacy	$72.5B	University research, ARC

a Information has been extracted from budget paper no. 1 and no. 4 and each portfolio’s budget statement.

This asymmetry appears structural rather than incidental. Health and Aged Care is the only portfolio that explicitly articulates the use of “evidence-based policy” tied to measurable outcomes across defined time horizons (1, 3–5). Portfolios of comparable scale, including Education and Defence, justify expenditure largely through informal mechanisms such as progress reporting, strategic alignment, and performance targets, with limited transparency regarding what constitutes sufficient evidence to warrant funding continuation or expansion (1, 3). The result is an uneven evaluative landscape where the Health portfolio faces the highest procedural and analytical burden to appropriate federal funding, thereby leading to a distorted opportunity cost trade-off decision making environment.

This imbalance now operates in tension with stated government objectives. The Federal Government has formally endorsed broader conceptions of value through the Measuring What Matters framework, which positions wellbeing, equity, sustainability, and social cohesion as core dimensions of policy success (6). However, Measuring What Matters is not procedurally embedded in Expenditure Review Committee (ERC) funding decisions (3–5, 7). Wellbeing is conceptually acknowledged but operationally absent at the point where funding is allocated, renewed, or withdrawn. The result is a rhetorical commitment to societal impact that remains disconnected from evaluative practice.

Against a backdrop of post-pandemic fiscal pressure, rising public debt, and demographic ageing, this asymmetry has become structurally unsustainable (8). Australia’s population is projected to age substantially over coming decades, with the proportion of people aged 65 years and over rising from approximately 15% in the mid-2010s to around 23% by the mid-2060s and continuing to increase thereafter (9). Given that per-capita health expenditure rises steeply with age, this compositional shift alone is expected to place upward pressure on total health spending (10). Health must justify programs within an environment of persistent demand growth driven by population ageing and chronic disease, while simultaneously meeting evidentiary standards that peer portfolios of similar fiscal scale need not satisfy. Without reform, fiscal constraint reinforces existing distortions, where the portfolio most committed to formal evaluation bears the greatest exposure to scrutiny, while others remain comparatively insulated.

## Scale does not predict scrutiny

Evidentiary standards across Commonwealth portfolios vary independently of fiscal scale. Health, Education, and Defence all operate at substantial budgetary levels, yet differ fundamentally in how value is articulated and assessed (Table 1) (1). In addition to the comparison mentioned previously between Health and Education, Defence is another consideration with its AU$83.2 billion in expenditure (1). Defence, while fiscally large, justifies expenditure through strategic alignment, capability arguments, and geopolitical necessity, with limited transparency regarding outcome valuation (1).

This divergence creates problematic system-level incentives. Portfolios with mature evaluative frameworks face higher administrative and analytical costs when proposing new initiatives or defending existing programs. Those without such frameworks encounter fewer procedural barriers. This diminishes the ability for Health to justify funding during ERC committee meetings. Evidence generation becomes unevenly rewarded, and the burden of proof falls disproportionately on portfolios that already engage most rigorously with evaluation.

The absence of shared evaluative baselines also constrains central decision-making. ERC members must adjudicate between submissions grounded in fundamentally different justificatory logics, with formal economic analyses alongside strategic narratives or qualitative claims. In such settings, trade-offs are resolved through negotiation, precedent, and political context rather than transparent comparison of societal impact. This reflects institutional design choices that allow heterogeneous standards to persist.

Importantly, this pattern cannot be attributed solely to differences in measurability. While some outcomes are inherently complex or long-term, the current observed variation between portfolios is from the inconsistently applied formal evaluative expectations rather than unavoidable technical constraints. The central insight is that fiscal scale does not predict scrutiny, despite the fact that larger expenditures entail greater opportunity cost and therefore ought to attract proportionately stronger justification.

## The measuring what matters gap

The introduction of Measuring What Matters signals explicit recognition that traditional economic indicators are insufficient to capture the full value of public investment (6). The framework establishes a conceptual precedent for assessing policy success through societal impact rather than narrow fiscal optimisation alone. It articulates five wellbeing themes supported by twelve dimensions and fifty indicators, drawing on national datasets and distributional analysis (6).

However, Measuring What Matters remains aspirational rather than operational. The framework is not formally embedded in ERC decision-making, nor does it carry procedural weight when funding proposals are assessed or renewed (3, 11). References to wellbeing themes are largely absent from portfolio submissions, and explicit integration with funding justification is rare outside Health and a small number of socially oriented portfolios (1).

This creates a critical implementation gap. Government rhetoric has shifted toward broader conceptions of value, yet the mechanisms required to operationalise this shift remain underdeveloped. In the absence of procedural integration, fiscal pressure defaults to reinforcing existing asymmetries. Health continues to bear the highest evidentiary burden precisely because it has the most developed evaluation infrastructure.

The question is no longer whether wellbeing should matter in budgeting, government policy statements have answered that affirmatively, but how wellbeing can be assessed in ways that are credible, proportionate, and comparable across portfolios. Without progress on this question, the gap between stated priorities and funding practice will widen, and evidence asymmetry will deepen.

## SROI as a bridging framework

Addressing evidence asymmetry does not require abandoning established methods. Cost–benefit analysis and health technology assessment remain essential, particularly within Health. However, these methods are not designed to capture the full range of social and wellbeing outcomes that governments increasingly claim to value, nor do they translate easily across portfolios with diffuse or long-term objectives (12).

Social Return on Investment (SROI) occupies a pragmatic middle ground. It offers structure and accountability without the rigidity or data demands of full cost–benefit analysis, allowing social and wellbeing outcomes to be articulated within a transparent evaluative framework (13). Importantly, SROI is complementary rather than substitutive, as it extends existing approaches (14). This distinction matters in policy contexts where resistance to methodological replacement can be high.

The proposed framework positions Social Return on Investment (SROI) as a valuation tool that raises the evidentiary floor for portfolios currently relying on narrative justifications, without displacing full economic evaluation where it already exists. In Australia’s current system, Health operates at the highest level of evidentiary standards, through cost-effectiveness, cost-utility and HTA processes that routinely generate quantifiable evidence of policy impact and fiscal value, for example through national analyses of PBS price-control policies demonstrating substantial reductions in pharmaceutical expenditure (15). This contrasts other portfolios (Education, Infrastructure, Social Services) which justify large expenditures via strategic alignment, performance indicators and descriptive program logic. This creates evidence asymmetry at the point where the Expenditure Review Committee (ERC) must adjudicate between submissions grounded in fundamentally different evidential logics. We therefore propose SROI functions as a bridging methodology that monetises non-market social outcomes and requires structured outcome and impact evidence, while stopping short of the data and modelling requirements of full HTA.

At the process level, SROI proposals would define clear programme boundaries, map stakeholder groups and their outcomes, quantify those outcomes using observable indicators, monetise them through transparent proxies, and present a conservative SROI ratio alongside sensitivity analysis. These programme-level SROI estimates would then feed into Portfolio Budget Statements and a central cross-portfolio evaluation registry, allowing ERC members to compare social value claims across portfolios on a more consistent basis.

Education and Infrastructure represent portfolios where SROI could introduce proportional evaluative standards without imposing the full analytical burden associated with formal economic appraisal. Outcomes in these portfolios such as human capital formation, equity, regional development, and environmental considerations, align closely with SROI’s conceptual foundations yet are currently assessed through informal or narrative mechanisms (1).

Health is uniquely positioned to lead this transition. The sector is already most aligned with SROI principles due to its mature evaluation infrastructure, established data systems, and familiarity with outcome-based assessment (1). Piloting SROI within Health represents a low-risk proof of concept, demonstrating how extra-welfarist outcomes can coexist with Treasury’s cost–benefit orientation (16, 17).

If successful, such a pilot could provide a template for broader application. SROI thus functions as a bridging framework connecting policy aspirations around wellbeing with practical evaluative practice in a manner that is both analytically credible and institutionally feasible.

## Governance and policy implications

The challenge is fundamentally one of governance. Australia’s budget system is highly effective at enforcing fiscal discipline but comparatively weak at ensuring consistency in how value is assessed across portfolios. While central agencies provide detailed guidance on costing, risk, and fiscal alignment, they do not prescribe how social value or evidence strength should be weighted across domains (3–5, 11).

International experience illustrates that alternative models are feasible. The UK’s use of coordinated appraisal and evaluation frameworks, supported by the Green Book, Magenta Book, and a central Evaluation Task Force, illustrates precedents that show central coordination can reduce opacity and improve cross-portfolio accountability (18, 19).

Incremental reform would likely be preferable to wholesale redesign. Clearer expectations for proportional evaluation, greater transparency regarding evidence thresholds, and the development of shared evaluative registries could reduce asymmetry without imposing uniform methods. The Freedom of Information Act 1982 also represents a practical mechanism for addressing opacity gaps in portfolios where evaluation frameworks are not publicly articulated.

For central agencies and the ERC, wellbeing frameworks must be operationalised and entrenched in guidance documents rather than merely articulated. Introducing complementary valuation frameworks alongside existing fiscal processes would help align rhetoric with practice and reduce evidence asymmetry over time. Health, by virtue of its existing evaluative maturity, is well positioned to act as the proof of concept for such reform.

## Conclusion

Evidence asymmetry sits at the core of how government justifies, evaluates, and renews expenditure. In an era of sustained fiscal constraint and demographic pressure, Health cannot continue to bear a higher burden of proof alone.

The emergence of Measuring What Matters creates a window of opportunity. Government has articulated broader conceptions of value but lacks mechanisms to operationalise them consistently. Without such mechanisms, fiscal pressure will reinforce existing inequities, penalising portfolios that already engage most rigorously with evaluation while leaving others insulated.

The question is whether government institutions are equipped to recognise wellbeing in practice, not just in principle. Aligning evidentiary standards with fiscal scale and societal impact is essential if public spending is to remain both credible and coherent. This requires more than rhetoric, and necessitates institutional reform that makes wellbeing assessable, comparable, and consequential in budget decisions.

## Data Availability

The original contributions presented in the study are included in the article/supplementary material, further inquiries can be directed to the corresponding author.
